# Effect of codeine on CYP450 isoform activity of rats

**DOI:** 10.1080/13880209.2017.1297466

**Published:** 2017-03-02

**Authors:** Shuanghu Wang, Yanwen Dong, Ke Su, Jing Zhang, Linyi Wang, Anyue Han, Congcong Wen, Xianqin Wang, Yan He

**Affiliations:** a The Laboratory of Clinical Pharmacy, The People's Hospital of Lishui, Lishui, China;; b Laboratory Animal Centre of Wenzhou Medical University, Wenzhou, China;; c Analytical and Testing Center of Wenzhou Medical University, Wenzhou, China;; d The Institute of Molecular Medicine, School of Optometry and Ophthalmology and Eye Hospital, Wenzhou Medical University, Wenzhou, China

**Keywords:** Cocktail, UPLC-MS/MS, bupropion, midazolam, biochemical

## Abstract

**Context:** Codeine, also known as 3-methylmorphine, is an opiate used to treat pain, as a cough medicine and for diarrhoea. No study on the effects of codeine on the metabolic capacity of CYP enzyme is reported.

**Objective:** In order to investigate the effects of codeine on the metabolic capacity of cytochrome P450 (CYP) enzymes, a cocktail method was employed to evaluate the activities of CYP2B1, CYP2D1, CYP1A2, CYP3A2 and CYP2C11.

**Materials and methods:** Sprague–Dawley rats were randomly divided into codeine group (low, medium, high) and control group. The codeine group rats were given 4, 8, 16 mg/kg (low, medium, high) codeine by continuous intragastric administration for 14** **days. Five probe drugs bupropion, metroprolol, phenacetin, midazolam and tolbutamide were given to rats through intragastric administration, and the plasma concentrations were determined by UPLC-MS/MS.

**Results and conclusion:** The pharmacokinetic parameters of bupropion and metroprolol experienced obvious change with AUC_(0-t)_, *C*_max_ increased and CL decreased for bupropion in medium dosage group and midazolam low dosage group. This result indicates that the 14** **day-intragastric administration of codeine may inhibit the metabolism of bupropion (CYP2B1) and midazolam (CYP3A2) in rat. Additional, there are no statistical differences for albumin (ALB), alkaline phosphatase (ALP), creatinine (Cr) after 14 intragastric administration of codeine, while alanine aminotransferase (ALT), aspartate aminotransferase (AST), uric acid (UA) increased compared to control group. The biomedical test results show continuous 14** **day-intragastric administration of codeine would cause liver damage.

## Introduction

Codeine, also known as 3-methylmorphine, is an opiate used to treat pain (Havig et al. 2016; Hudak 2016; Kimergard et al. 2016), as a cough medicine, and for diarrhoea (Prommer 2011). It is typically used to treat mild to moderate degrees of pain. Codeine is also used to treat diarrhoea and diarrhoea-predominant irritable bowel syndrome, although loperamide, diphenoxylate, paregoric or even laudanumare more frequently used to treat severe diarrhoea. There is weak evidence that it is useful in cancer pain but it is associated with increased side effects. Common adverse effects associated with the use of codeine include drowsiness and constipation. As with all opiates, longer-term effects can vary, but can include diminished libido, apathy and memory loss. Some people may also have an allergic reaction to codeine, such as the swelling of skin and rashes.

Cytochrome P450 (CYP) enzymes are responsible for most biotransformation steps of xenobiotics and endogenous molecules (Chen et al. 2016). They are a large and diverse group of enzymes, originally found in liver microsomes. Currently, 17 categories of CYP gene families including a total of 86 CYP genes have been found (Saad et al. 2016). Among these CYP families, 57 CYP enzymes encoded in the human genome, only around 15 CYP enzymes are involved in metabolism and only five CYPs account for 95% all marketed drugs (Guengerich 2008). Variations of their activity by inhibition or induction can influence the pharmacokinetics and thereby the effect of drugs (of abuse) (Geng et al. 2015; Li et al. 2015). Enzyme inhibition by co-administered drugs (of abuse) and/or genetic variations of their expression can increase the risk of adverse reactions or reduce the desired effect (Derungs et al. 2016). Such drug–drug interactions were described as a major reason for hospitalization or even death (Dinger et al. 2014).

So far, no study on the effects of codeine on the metabolic capacity of CYP enzyme has been reported. Therefore, in this study, five probe drugs were employed to evaluate effect of codeine on the metabolic capacity of five important hepatic drug metabolic enzymes, CYP2B1, CYP2D1, CYP1A2, CYP3A2, CYP2C11. The effects of codeine on rat CYP enzyme activity will be evaluated according to the pharmacokinetic parameters changes of five specific probe drugs (bupropion, metroprolol, phenacetin, midazolam and tolbutamide).

## Materials and methods

### Chemicals

Bupropion, metroprolol, phenacetin, midazolam and tolbutamide (all >** **98%) and the internal standard diazepam (IS) were obtained from Sigma-Aldrich Company (St. Louis, MO). Ultra-pure water was prepared by Millipore Milli-Q purification system (Bedford, MA). Methanol and acetonitrile (HPLC grade) were obtained from Merck Company (Darmstadt, Germany).

### Animals

Sprague–Dawley rats (male, 220** **±** **20** **g) were purchased from Shanghai SLAC Laboratory Animal Co., Ltd. Animals were housed under a natural light-dark cycle conditions with controlled temperature (22** **°C). All 28 rats were housed at Laboratory Animal Research Center of Wenzhou Medical University. All experimental procedures were approved ethically by the Wenzhou Medical University Administration Committee of Experimental Animals.

### Pharmacokinetics

Twenty-eight rats (220** **±** **20** **g) were randomly divided into four different dosages of codeine groups (low, medium, high dosages and control group with seven rats in each group). Three different codeine group (low-group, medium-group, high-group) were given codeine 4, 8, 16** **mg/kg one time by intragastric administration at every morning, respectively, and last for 14** **days. Control group were given saline by same administration method. At 15** **days morning, five probe drugs bupropion, metroprolol, phenacetin, midazolam and tolbutamide were mixed in corn oil and given to the rats of three codeine groups and control group by intragastric administration at a single dosage 10** **mg/kg for bupropion, metroprolol, phenacetin, midazolam, 0.1** **mg/kg for tolbutamide.

Blood (0.3** **mL) samples were collected into heparinized 1.5** **mL polythene tubes from the tail vein at 0.0833, 0.5, 1, 2, 3, 4, 6, 8, 12, 24** **h after intragastric administration of five probe drugs. Hundred microliters of plasma was obtained from blood sample after centrifugation at 4000** ***g* for 10** **min. In a 1.5** **mL centrifuge tube, 200** **μL of acetonitrile (containing 50** **ng/mL IS) was added into 100** **μL of collected plasma sample. After vortex-mixing for 1.0** **min, the sample was centrifuged at 13,000** ***g* for 15** **min. Then the supernatant (2** **μL) was injected into the UPLC-MS/MS system for analysis.

### UPLC-MS/MS determination of probe drugs

**Figure 1.**
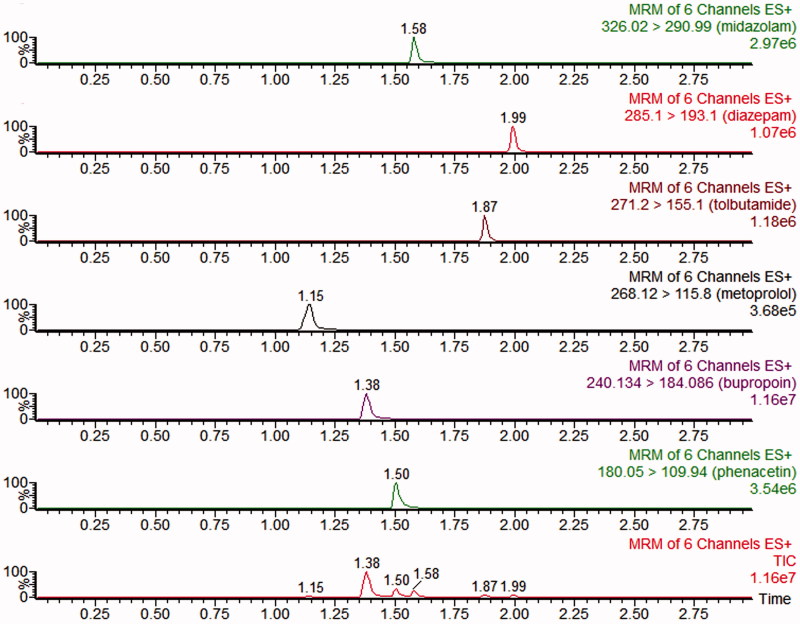
UPLC-MS/MS chromatograms, blank plasma spiked with midazolam, tolbutamide, metroprolol, bupropion, phenacetin and diazepam (IS).

The concentration of bupropion, metroprolol, phenacetin, midazolam and tolbutamide in rat plasma were simultaneously determined by a sensitive and simple UPLC-MS/MS method (Ma et al. 2015). The probe drugs were analyzed by a UPLC-MS/MS with ACQUITY I-Class UPLC and a XEVO TQD triple quadrupole mass spectrometer that equipped with an electrospray ionization (ESI) interface (Waters Corp., Milford, MA). UPLC-MS/MS chromatogram (Figure 1), blank plasma spiked with midazolam, tolbutamide, metroprolol, bupropion, phenacetin and diazepam (IS). The LLOQ for each probe drug in plasma was 2** **ng/mL. The RSD of the five probe drugs were less than 15%. The calibration plot of the probe drugs was in the range of 2–2000** **ng/mL (*r*** **>** **0.995).

### Biochemical tests

After the pharmacokinetic study, the blood was collected from the tail vein for biochemical tests of serum alanine aminotransferase (ALT), aspartate aminotransferase (AST), albumin (ALB), alkaline phosphatase (ALP), creatinine (Cr), uric acid (UA). Serum samples were analyzed to measure the serum activities of ALT, AST, ALP, Urea, Cr and UA, which was used to evaluate the liver and kidney function.

### Data analysis

Concentration of plasma probe drugs versus time was analyzed by Version 3.0 Data Analysis System (Wenzhou Medical University, China). The main pharmacokinetic parameters and biochemical results of the codeine group and control group were analyzed by SPSS l8.0 statistical software; statistical significance was assessed by *t*-test (*p* < 0.05 was considered as statistically significant).

## Results

### Pharmacokinetics

**Figure 2.**
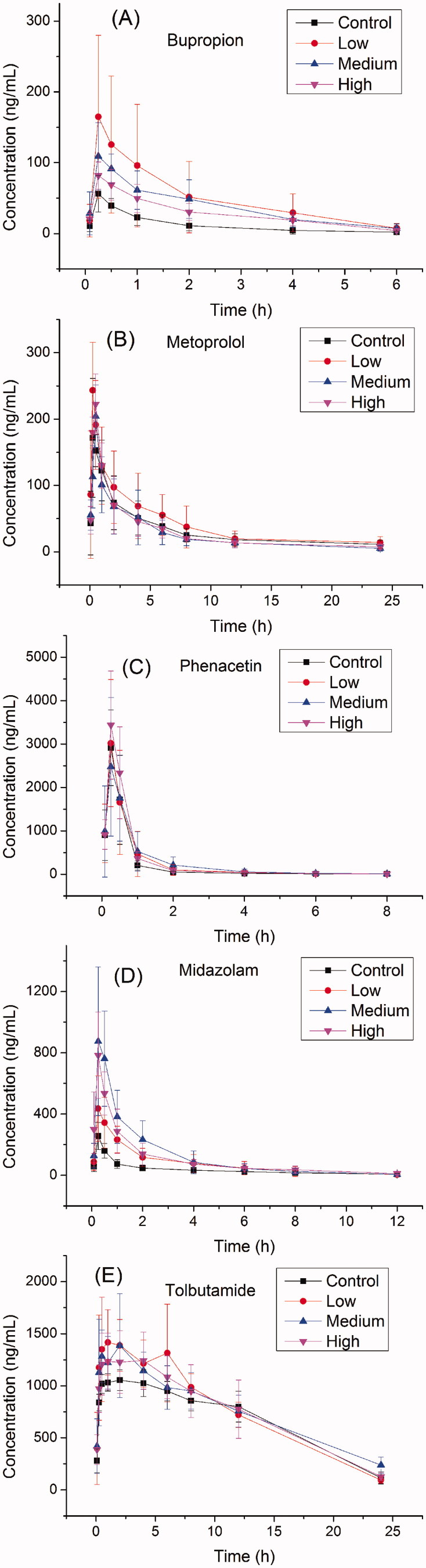
The pharmacokinetic profiles of bupropion, metroprolol, phenacetin, midazolam, tolbutamide in control group and codeine group (low, medium, high) rats (*n*** **=** **8).

The main pharmacokinetic parameters of bupropion, metroprolol, phenacetin, midazolam and tolbutamide calculated from non-compartment model analysis were summarized in Table 1. The representative profiles of concentration of drugs (bupropion, metroprolol, phenacetin, midazolam and tolbutamide) versus time were presented in Figure 2.

**Table 1.** Pharmacokinetic parameters of probe drugs from control group and codeine group rats (mean** **±** **SD, *n*** **=** **8).

Parameters	AUC _(0-t)_ ng/mL*h	AUC _(0-∞)_ ng/mL *h	*t*_1/2z_ h	CLz/F L/h/kg	Vz/F L/kg	*C*_max_ ng/mL
Bupropion (CYP2B1)						
Control	68.7 ± 39.4	71.0 ± 41.1	0.8 ± 0.3	174.4 ± 74.8	184.0 ± 41.4	57.6 ± 24.1
Low	294.5 ± 256.3*	306.7 ± 265.0*	1.1 ± 0.3	58.7 ± 46.3**	87.1 ± 60.9**	166.4 ± 113.3*
Medium	224.4 ± 81.5**	247.3 ± 98.4**	1.6 ± 0.6*	46.5 ± 18.6**	99.3 ± 28.1**	116.1 ± 41.3**
High	170.0 ± 45.4**	191.4 ± 61.0**	1.8 ± 0.6**	57.5 ± 20.2**	144.5 ± 56.1	90.9 ± 16.9*
Metroprolol (CYP2D1)						
Control	768.5 ± 262.3	888.3 ± 314.7	8.0 ± 5.1	13.5 ± 7.8	131.7 ± 63.2	197.8 ± 65.6
Low	977.8 ± 484.9	1065.2 ± 529.6	5.0 ± 1.5	10.8 ± 4.0	72.0 ± 11.8	243.9 ± 72.2
Medium	642.4 ± 243.1	710.0 ± 259.5	7.5 ± 3.6	16.5 ± 8.8	163.9 ± 72.8	204.3 ± 47.0
High	701.2 ± 168.9	833.3 ± 253.8	9.6 ± 7.0	13.4 ± 5.8	158.6 ± 86.0	222.5 ± 45.5
Phenacetin (CYP1A2)						
Control	1649.2 ± 662.8	1658.2 ± 662.7	1.5 ± 0.5	7.2 ± 3.6	16.4 ± 10.6	2967.2 ± 899.2
Low	1979.4 ± 1267.0	1992.7 ± 1266.8	1.4 ± 0.5	7.3 ± 5.0	15.0 ± 10.0	3025.8 ± 1456.5
Medium	2163.0 ± 1339.3	2178.8 ± 1337.1	1.3 ± 0.7	6.6 ± 4.5	15.1 ± 20.5	2542.0 ± 1567.8
High	2197.0 ± 780.3	2204.0 ± 778.0	1.2 ± 0.4	5.0 ± 1.4	8.8 ± 4.2	3438.6 ± 1244.9
Midazolam (CYP3A2)						
Control	16559.4 ± 1541.1	18827.3 ± 3083.7	7.4 ± 3.6	0.054 ± 0.009	0.552 ± 0.169	1108.1 ± 113.0
Low	18299.6 ± 1854.6	18990.4 ± 1710.4	4.8 ± 0.9	0.053 ± 0.005	0.368 ± 0.083*	1708.0 ± 395.5*
Medium	18352.5 ± 2074.6	21189.1 ± 3100.1	8.4 ± 2.2	0.048 ± 0.007	0.570 ± 0.113	1511.0 ± 476.5
High	17933.0 ± 4145.6	19414.2 ± 4406.4	6.3 ± 2.0	0.055 ± 0.018	0.473 ± 0.131	1294.7 ± 304.2
Tolbutamide (CYP2C11)						
Control	16559.4 ± 1541.1	18827.3 ± 3083.7	7.4 ± 3.6	0.054 ± 0.009	0.552 ± 0.169	1108.1 ± 113.0
Low	18299.6 ± 1854.6	18990.4 ± 1710.4	4.8 ± 0.9	0.053 ± 0.005	0.368 ± 0.083*	1708.0 ± 395.5*
Medium	18352.5 ± 2074.6	21189.1 ± 3100.1	8.4 ± 2.2	0.048 ± 0.007	0.570 ± 0.113	1511.0 ± 476.5
High	17933.0 ± 4145.6	19414.2 ± 4406.4	6.3 ± 2.0	0.055 ± 0.018	0.473 ± 0.131	1294.7 ± 304.2

(Codeine group was compared with the control group, *p < 0.05, **p *<* 0.01).

From Table 1, no difference in pharmacokinetic behaviours can be observed between codeine group and control group for metroprolol, phenacetin and tolbutamide. Table 1 shows the pharmacokinetic behaviours of bupropion in codeine group compared with the control group, AUC_(0-t)_ increased (*p* < 0.01, *p*** **<** **0.05), CL decreased (*p* < 0.01, *p*** **<** **0.05), *C*_max_ increased (*p* < 0.01), and the similar results were found for midazolam.

### Biochemical tests

There are no statistical differences for albumin (ALB), alkaline phosphatase (ALP), creatinine (Cr) after 14 intragastric administration of codeine, while alanine aminotransferase (ALT), aspartate aminotransferase (AST), uric acid (UA) increased compared to control group, Table 2. The biomedical test results show continuous 14** **days-intragastric administration of codeine would cause liver damage.

**Table 2.** Biochemical results in rat serum after intragastric administration of codeine in the fourteenth day (mean** **±** **SD, *n*** **=** **8).

Group	Alanine aminotransferase (ALT)	Albumin (ALB)	Aspartate aminotransferase (AST)	Alkaline phosphatase (ALP)	Creatinine (Cr)	uric acid (UA)
Control	39	23	180	165	36	80
Low	60	26	430*	134	38	293**
Medium	76**	25	491**	153	33	245*
High	57*	27	368*	181	38	273*

Compared control group with alprazolam treated group (4, 8, 16 mg/kg, low, medium, high),

* *p*** **<** **0.05 and **p** **<** **0.01, as indicated by the statistical analysis *T*-test.

## Discussion

There are no significant differences for AUC, CL and *C*_max_ of metroprolol, phenacetin and tolbutamide between the codeine group and control group. The codeine was not able to induce or inhibit the activity of metroprolol (CYP2D1), phenacetin (CYP1A2) and tolbutamide (CYP2C11) enzyme.

The pharmacokinetic parameters of bupropion and midazolam experienced obvious change with increased AUC_(0-t)_, *C*_max_ and decreased CL for in bupropion in medium dosage group and midazolam low dosage group. This result indicates that the 14** **day-intragastric administration of codeine may inhibit the metabolism of bupropion (CYP2B1) and midazolam (CYP3A2) in rat. However, additional studies for the effect of codeine activity of CYP2B1 and CYP3A2 should be done.

As codeine is always administered in combination with other drugs, interactions between codeine and other drugs would increase the risk of either diminished efficacy or adverse effects (Kathiramalainathan et al. 2000; Romach et al. 2000; Wilcox & Owen 2000; Fernandes et al. 2002; Madadi et al. 2011; Crews et al. 2012; Frost et al. 2012; Andresen et al. 2013; Asturias-Arribas et al. 2014; Crews et al. 2014). In our study, we found that 14 days of intragastric administration of codeine inhibit the CYP2B1 and CYP3A2. Therefore, the metabolism and elimination of drugs would change if they are administrated in combination with codeine. While biomedical test results show continuous 14** **day intragastric administration of codeine would cause liver damage.

According to published studies, CYP1A1, 1A2, 2B1, 2C11 and 3A2 in rat CYP1A1, 1A2, 2B6, 2C9 and 3A4 proteins in and human have 78, 70, 74, 77 and 73% homology, respectively (Wiseman & Lewis 1996). Moreover, rat CYP2B1 and CYP3A2 were different with CYP1A1 and 2C6 which have no gender difference, CYP2B1 is male dominant, and CYP3A2 is male specific (Zhu et al. 2007). Therefore, if the metabolism of CYP2B1 and CYP3A2 was potentially inhibited by codeine in rat, there would be also inhibition of CYP2B1 and CYP3A2 in humans who taken codeine for a long time. The metabolism and elimination of drugs would change if they are administrated in combination with codeine.

Although there were only pharmacokinetic parameters changed, and it’s inadequate and unsuitable to make an affirmative conclusion, the results of the inhibition of bupropion (CYP2B1) and midazolam (CYP3A2) in rat can give some inspiration for us, and the further study for the effect of codeine activity of CYP2B1 and CYP3A2 should be done.

In conclusion, the results observed in this study would provide us valuable information regarding the interactions of codeine with other drugs. Continuous 14** **day intragastric administration of codeine may inhibit the activities of CYP2B1 and CYP3A2 of rats. Inhibition of drug metabolizing enzyme would increase the concentration of other drugs which were metabolized by CYP2B1 and CYP3A2.
